# Is well-becoming important for children and young people? Evidence from in-depth interviews with children and young people and their parents

**DOI:** 10.1007/s11136-023-03585-w

**Published:** 2024-01-31

**Authors:** Samantha Husbands, Paul Mark Mitchell, Philip Kinghorn, Sarah Byford, Cara Bailey, Paul Anand, Tim J. Peters, Isabella Floredin, Joanna Coast

**Affiliations:** 1 Health Economics Bristol, Population Health Sciences, Bristol Medical School, University of Bristol, Bristol, BS8 1NU UK; 2 Population Health Sciences, Bristol Medical School, Bristol Dental School, University of Bristol, Bristol, BS1 2LY UK; 3 Health Economics Unit, Institute of Applied Health Research, University of Birmingham, Birmingham, B15 2TT UK; 4 King’s Health Economics, Institute of Psychiatry, Psychology & Neuroscience at King’s College London, London, SE5 8AF UK; 5 Economics, The Open University, Milton Keynes, UK; 6 Department of Social Policy and Intervention, Oxford University, Oxford, UK; 7 Centre for Philosophy of Social and Natural Sciences, London School of Economics, London, UK; 8 School of Nursing, Institute of Clinical Sciences, College of Medical and Dental Sciences, University of Birmingham, Birmingham, B15 2TT UK

**Keywords:** Children and young people, Economic measure development, Wellbeing measures, Well-becoming, Capabilities

## Abstract

**Purpose:**

This study explores how important well-becoming factors appear to be to children during childhood. We define well-becoming as the indicators which predict children and young people’s future wellbeing and opportunities. The priority for this work was to explore whether well-becoming might be an important factor to include in outcome measures for children and young people. The inclusion of well-becoming indicators could ensure that opportunities to invest in promoting wellbeing in children’s futures are not missed.

**Methods:**

In-depth, qualitative interviews (*N* = 70) were undertaken with children and young people aged 6–15 years and their parents. Analysis used constant comparison and framework methods to investigate whether well-becoming factors were considered important by informants to children and young people’s current wellbeing.

**Results:**

The findings of the interviews suggested that children and young people and their parents are concerned with future well-becoming now, as factors such as future achievement, financial security, health, independence, identity, and relationships were identified as key to future quality of life. Informants suggested that they considered it important during childhood to aspire towards positive outcomes in children and young people’s futures.

**Conclusion:**

The study findings, taken alongside relevant literature, have generated evidence to support the notion that future well-becoming is important to current wellbeing. We have drawn on our own work in capability wellbeing measure development to demonstrate how we have incorporated a well-becoming attribute into our measures. The inclusion of well-becoming indicators in measures could aid investment in interventions which more directly improve well-becoming outcomes for children and young people.

**Supplementary Information:**

The online version contains supplementary material available at 10.1007/s11136-023-03585-w.

## Introduction

Comparing the costs and outcomes of health and social care interventions via economic evaluation plays an important role in the allocation of scarce resources [1, 2]. In health economics, this has mostly involved the development and use of measures focused on health-related quality of life (HRQOL) used to generate Quality Adjusted Life Years (QALYs) [3]. However, broader wellbeing measures, particularly those focused on capability wellbeing [4], meaning an individual’s ability to be and do the things in life that are of value to them [5], have become viable alternatives in health and social care decision-making [6, 7]. Focusing on children and young people (CYP), this broader approach to outcome measurement is helpful, given that health gain is only likely to be one of many influential factors important to CYP’s wellbeing [8].

Measures developed for CYP should be relevant and sensitive to changes in CYP’s quality of life [9] and thus developed specifically with CYP populations [10, 11]. This said, developing measures with and for CYP is challenging, including issues around the collection of primary data from CYP to inform measure attributes [12] and with the valuation of attributes given the complexity of valuation tasks [13]. This paper will focus on the broader challenge of determining whether measures for CYP should focus only on current wellbeing or take account of the potential for well-becoming also, with well-becoming defined as the indicators which predict CYP’s future wellbeing and opportunities [14].

Previous health economic CYP measures have not openly considered well-becoming, instead focusing on current quality of life status [15]. This is in line with broader literature on child welfare measurement, which has predominantly shifted in focus towards the importance of measuring and enhancing CYP’s current wellbeing and away from the previous norm of conceptualising child wellbeing in terms of children’s futures [16]. Previous studies often focused on evaluating CYP’s welfare by predicting anticipated wellbeing during adulthood, on specific indicators such as future employability [14, 17]. However, it was argued that a focus only on well-becoming ignores childhood as an explicit stage of life, viewing CYP as future adults rather than present day citizens, thus potentially missing opportunities to evaluate and improve wellbeing during childhood [16, 17].

Accepting the importance of measuring and looking to improve CYP’s current wellbeing through outcome measurement does not mean that we should ignore well-becoming altogether. A recent paper has advocated for the importance of well-becoming in health economics research and specifically in economic evaluation, suggesting that a shift is needed to a “prevention agenda” to improve health and social problems [18]. Indeed, Nobel prize winning economist James Heckman argues that investment during childhood in programs which foster future capabilities is important to improving outcomes in adulthood and reducing inequalities for disadvantaged CYP [19]. Heckman states that building capabilities important to future wellbeing early on in life is key to the continuation of these capabilities into the future; for example, investment to increase motivation in childhood will bring about continued motivation in adulthood [19]. This notion fits well with the work of Caroline Hart (2016), who suggests that CYP’s aspirations during childhood are likely to be indicative of what they strive to achieve as adults [20]. Hart (2016) refers to this as “capability to aspire” and suggests that a lack of investment in CYP’s aspirations during childhood can impact opportunities for future flourishing [20]. This presents an argument for including well-becoming alongside wellbeing in child measures [21], with a view to additionally capturing and improving factors important to CYP’s future outcomes. Incorporating aspects of wellbeing and well-becoming in measure development could potentially ensure that opportunities to invest in promoting wellbeing in CYP’s futures are not missed through a focus just on current wellbeing.

As part of a large-scale project aimed at developing capability wellbeing outcome measures for CYP [22], this paper uses qualitative data collected from CYP and their parents to explore how important well-becoming factors—or more broadly CYP’s futures—appear to be to CYP during childhood. Analysis focuses on whether future well-becoming, including desires and concerns for the future, is considered important to CYP now, with an aim to generate evidence that could support incorporating well-becoming outcomes into measures for CYP. The paper presents the methods and results for this empirical study, and in the discussion, we present the implications of including well-becoming attributes in child measures, with insight into how we are incorporating well-becoming into our own CYP outcome measure development.

## Methods

### Sampling

CYP aged 6–15 years and their parents were sampled predominantly from South West England, but also from other areas to enhance representativeness, including West Midlands, East Midlands, London, North East and South East England. Purposeful maximum variation sampling [23] was employed to sample informants of different ages, genders and from different ethnic, socioeconomic, and family backgrounds (one and two parent families). Our sampling approach focused on ensuring that the key characteristics of the general CYP population were represented and aimed to be as broad as possible to potentially capture any differences in views about what was important to CYP in terms of well-becoming. Socioeconomic backgrounds of CYP were determined using the English Index of Multiple Deprivation (IMD) measure, which ranks small areas in England from most deprived to least deprived in ten equal deciles [24]. We also aimed to recruit CYP from urban and rural areas, and those with any health condition. Potential informants were only excluded if they were unable to understand the study information. Snowball sampling [25] was used to sample siblings of CYP taking part. Informants were initially sampled through schools and charitable organisations. A study invitation letter, screening questionnaire and information sheet were sent to parents from the school or charitable organisation. If parents were happy for their child to participate in the study, they completed the screening questionnaire and returned it to the research team. Interviews were arranged directly by the research team or by the educational/charitable organisation.

Later in the study, in response to recruitment difficulties because of COVID-19 [26], informants were sampled and recruited online using Facebook. A Facebook page was set up and a Facebook ‘post’ created to summarise the study and to appeal for informants. This post was targeted at parents of CYP aged 6–15 years and was shared using the Facebook targeted Ads feature, for which Facebook charged a nominal fee. Parents were asked to contact SH if they were interested in participation. SH sent interested parents a study information sheet, and later, the study questionnaire if they wanted to participate. Interviews were arranged with parents directly.

Parents were asked to provide informed consent for all CYP and for their own participation. CYP aged 6–15 years were asked to provide written ‘assent’ for their involvement. The study was granted ethical approval by the University of Bristol Faculty of Health Sciences Research Ethics Committee (reference 77,121).

### Data collection

All interviews were undertaken by SH. In-depth interviews were initially undertaken with CYP, and their parents separately either in their own home or in the educational or charitable organisation. Interviews after the outbreak of COVID-19 were carried out online using Microsoft Teams. The decision regarding whether CYP were interviewed alone or with parents or other adults present was made in conjunction with the CYP, the parent and/or the educational/charitable organisation. If adults were present, they were asked not to answer on behalf of CYP.

CYP informants were asked to complete a hierarchical mapping activity [27] before the interview. They were asked to think of things that were important to them and draw or write these on sticky notes and arrange the completed sticky notes around a photograph or drawing of themselves. The interview focused on asking the CYP about the things they had recorded as important and why. Parents were questioned on what they thought enhanced and negatively impacted their child’s happiness. Both groups of informants were also asked about what they wanted for the future. Topic guides for the interviews are available in Online Appendix 1 and an example of a completed hierarchical mapping activity in Online Appendix 2.

### Data analysis

Interviews were audio-recorded and transcribed verbatim. Interview analysis used methods of constant comparison, whereby the meaning of new and existing data categories are continually compared to develop a deeper understanding of new and existing themes [28]. Transcripts were coded line-by-line and representative labels assigned to data to summarise meaning. Open coding [29] was initially used to identify mentions of CYP’s futures and factors considered important to future well-becoming. Coding became increasingly hierarchical as relationships between data categories became established and higher-level categories could be used to link lower-level codes in terms of the overarching concepts important to CYP’s well-becoming. Analysis of interview transcripts was carried out iteratively to allow new and developed categories to be applied to future transcripts. Analytic accounts were created to describe emerging data in context and to compare responses of informants under each data category [30]. Developing analytic accounts helped to further define data categories and facilitated an understanding of the relationships between categories, aiding the move to hierarchical coding and eventually generating the overall factors considered important to CYP’s well-becoming. Separate analytic accounts were developed for informant groups to take account of any potential differences in what was considered important to well-becoming. Informant groups were defined by the school age groupings most used within England: primary school aged CYP (aged 6–10 years), parents of primary school aged CYP (herein referred to as primary parents), secondary school CYP (aged 11–15 years) and parents of secondary school aged CYP (herein referred to as secondary parents). Discussion within accounts focused on similarities and differences in what informants considered to be important to CYP’s future quality of life. The framework method [31] was used to chart the responses of informants and to look for patterns in categories related to well-becoming. Using Excel, well-becoming categories were charted in the top horizontal axis, with each informant listed in the vertical axis, and informant quotations were recorded where they referred to a well-becoming factor. The analytic accounts and framework were taken together to consider how well-becoming factors had been expressed as important by CYP and parents.

## Results

Seventy in-depth interviews were undertaken with CYP aged 6 to 10 years (*n* = 24), primary parents (*n* = 13), CYP aged 11 to 15 years (*n* = 19) and secondary parents (*n* = 14). CYP informants were represented across the entire age range, with a balanced divide between male and females. Although most CYP informants were white British (*n* = 31) and from urban areas (*n* = 34), there was representation from those from other ethnic backgrounds (*n* = 12) and living in rural areas (*n* = 9). All parent informants lived with the CYP who they were discussing. Informant numbers and CYP characteristics are given in Table 1, 2 and 3. Primary school aged CYP interviews lasted between 18 and 45 min and primary parent interviews between 31 and 58 min. Secondary school aged CYP interviews lasted between 16 and 60 min and secondary parents between 26 and 51 min. The next sections provide a summary of the analysis of CYP and parent responses in relation to the well-becoming elements considered important to CYP’s quality of life. These are arranged according to whether they were raised as important by parents and CYP or by parents only.

**Table 1:** CYP and parent informant numbers

Informant	Number
Primary aged CYP (aged 6 to 10 years)	24
Secondary aged CYP (aged 11–15 years)	19^a^
Parents/guardians of primary aged CYP	13
Parents/guardians of secondary aged CYP	14

^a^This number includes the perspective of one secondary parent who acted as a proxy for her 12-year-old daughter due to her having severe learning difficulties and being unable to participate in the study directly

**Table 2:** Characteristics of CYP sample aged 6–10 years

CYP characteristics	CYP sample total	Associated parents^a^
*Age (years)*		
6	4	3
7	3	3
8	4	3
9	6	5
10	7	5
*Gender*		
Male	10	7
Female	14	12
*Ethnicity*		
White British	18	17
Black	3	0
Mixed	1	1
Asian	1	1
Asian Bangladeshi	1	0
*Deprivation level*^b^		
1–3 (most deprived)	4	0
4–7	10	10
8–10 (least deprived)	10	9
*Urban/Rural*		
Urban	20	13
Rural	4	6
*Health condition*		
Health condition	6	5
No health condition	18	14
*Family*		
One parent	5	2
Two parents	19	17

^a^Numbers of associated parents in the final column exceed the total number of parents in the sample because some parents were interviewed about multiple CYP

^b^Deprivation level determined using the English Index of Multiple Deprivation (IMD), which ranks small areas in England from most deprived to least deprived in ten equal groups (with IMD decile 1 being most and decile 10 being least deprived

**Table 3:** Informant characteristics of CYP sample aged 11–15 years

CYP Characteristics	CYP sample total	Associated parents^a^
*Age (years)*		
11	4	4
12	7^b^	6
13	2	1
14	4	4
15	2	1
*Gender*		
Male	10	7
Female	9	9
*Ethnicity*		
White British	13	12
Black	1	0
Asian	4	4
Other	1	0
*Deprivation level*^c^		
1–3 (most deprived)	5	2
4–7	8	8
8–10 (least deprived)	6	6
*Urban/Rural*		
Urban	14	11
Rural	5	5
*Health condition*		
Health condition	4	4
No health condition	15	12
*Family*		
One parent	6	4
Two parents	13	12

^a^Numbers of associated parents in the final column exceed the total number of parents in the sample because some parents were interviewed about multiple CYP

^b^This number includes the perspective of one secondary parent who acted as a proxy for her 12-year-old daughter due to her having severe learning difficulties and being unable to participate in the study directly

^c^Deprivation level determined using the English Index of Multiple Deprivation (IMD) measure, which ranks small areas in England from most deprived to least deprived in ten equal groups (with IMD decile 1 being most and decile 10 being least deprived)

### Well-becoming factors important to CYP and parents

#### Achievement now for the future

There was discussion from informants in all groups about achieving now for the future, with emphasis on educational achievement. Several CYP in the primary and secondary age groups spoke about going to school and getting an education, specifically being able to progress into higher education and getting a job. For some of these informants, achieving in school now was specifically about learning skills and getting the academic qualifications needed for their desired careers.

PC039 (primary CYP aged 10): *“…GCSEs and exams…you need to know it…[It’s important to] where you’re going to work….”*

PC106 (secondary CYP aged 14): “*The field of work I want to go into, you can’t get into it without school.”*

However, for some other CYP, educational achievement related to broader future aspirations, with informants commenting that they wanted to achieve a well-paid and good placing/high status job, indicting a desire for future financial security. All these informants were in the primary age group and over half of them were from the most deprived socio-economic groups.

PC004 (primary CYP aged 10): *“….If you don’t get an education, you’re more likely not to get money and you won’t be able to pay something in the future...”*

PC023 (primary CYP aged 8) “*I’m going to pass university and…have a good job…I don’t want to mess about and be poor….”*

Almost half of parents, across the primary and secondary groups, shared a similar view to these CYP on the importance of educational achievement now for the future, suggesting that getting a good education facilitated ongoing educational attainment and achieving a ‘good’ career and/or their career choice.

PA089 (primary parent):* “…. education…university. She says she wants to be a vet, and I would love nothing more than for her to achieve that….”*

#### Future physical and emotional security

Future physical security, referring to CYP’s future capability to afford food and shelter was raised by secondary parents and some (mostly secondary-aged) CYP. CYP suggested that they were concerned about being able to afford a house and food in the future. Of the CYP mentioning future security as important, over half were from the most deprived socio-economic backgrounds.

PC034 (secondary CYP aged 12): *“….I’ve heard so many stories of people not being able to afford houses because of inflation…I just hope that I’ll be able to afford a house.”*

PC043 (secondary CYP aged 11): *“…you get money, and you can feed yourself and look after yourself…As long as you have food and a home, that’s really all you need….”*

The views of the above CYP aligned with several secondary parents who were concerned for their children’s future security in terms of them having secure finances to afford a house and meet other needs. These parents were not related to the CYP above.

PA031 (secondary parent): *“….financial burdens and things ….It’s so expensive….I do save for them every week… I doubt it will be enough for a deposit on a house…”*

PA036 (secondary parent): *“…I think financial, and job, security are going to be important…There are so many jobs that are zero hours contracts…I’m really worried….”*

Other comments on future physical security referred to the importance parents placed on future health and focused on protecting future good health by promoting healthy behaviours in childhood. Parents discussed encouraging their child to exercise and/or eat a healthy and balanced diet now to promote good health in the future. The importance of future health was mentioned mostly by primary parents but also a minority of secondary parents:

PA091 (primary parent): *“…part of it is just learning self-discipline…It’s rules for as you get older…learn how to have that balance to try and keep healthy and happy.”*

PA017 (primary parent): *“…I read somewhere, if they do gymnastics when they’re little…it makes their joints more open and more able to do things as they get older.….they’ll be conscious that they like to move their body.”*

The importance of future emotional security was raised by a few secondary parents, with two suggesting that emotional security during childhood in the form of secure relationships with those around them will help them to feel secure in themselves and in their relationships in the future.

PA112 (secondary parent)**:** “*if you’ve tested out and you know what strong bonds are…that gives you the confidence in any new relationship…”*

PA037 (secondary parent): *“…social relationships…that confidence...to…speak to people…It’s important for them to feel a part of society, and to feel that they fit somewhere.”*

#### Future attachment

The importance of future attachment was raised in both parent groups and in some (mostly secondary) CYP interviews. The CYP suggested that future relationships were important to them, mentioning long-term friendships but mostly talking about having a family of their own.

PC109 (secondary CYP aged 12): *“Getting married, having children…I want that…”*

PC111 (secondary CYP aged 14): “*...to have my own children. I’d like to settle down and have a family….”*

Parents also talked about wanting their child to have fulfilling relationships in the future, including with their siblings, friends, and eventually having their own family. Some of these parents focused on the importance of their child socialising and developing social skills now to form the basis of their social relationships in the future.

PA110 (secondary parent):* “…people her own age that she can relate to…and hopefully those friendships will last well into adulthood…”*

PA011 (primary parent): *“[friendship] gives him confidence about himself and…social skills. And practising social skills, really, with the safe people that are in his life….”*

#### Future identity

Developing identity was raised across both parent groups and in the accounts of several primary and secondary aged CYP. CYP’s discussion of future identity centred on what they would like to do in future careers, with expressions of identity, such as wanting to be someone who helped others, seemingly entering their reasoning.

PC032 (secondary CYP aged 12): *“I want to be a doctor….it’s about helping people…”*

PC038 (secondary CYP aged 13): *“I really want to be a nurse…talking to people about if they have a problem and helping them.”*

Parents suggested it was important for CYP to develop a sense of who they are and what they like during childhood, helping them to feel confident in themselves and identify what will make them happy in adulthood.

PA036 (secondary parent): “*…He’s got to decide what his sexuality is or whether he gets married….I don’t have a vision of what we’re trying to turn him into beyond somebody who can…feel comfortable with who he is…..”*

PA100 (secondary parent): *“As a child…you should have a go at lots of different things and discover what you actually like…. He [CYP] might…be an old man in his shed with his woodworking tools and talk about when he was 11, he was bought a plane….”*

### Well-becoming factors important just to parents

#### Future independence

The importance of independence was raised across both parent accounts, with the view being that it was important to give CYP a level of independence to prepare them for being independent adults in the future. This included giving them the capability to deal with situations and make judgements and decisions independently.

PA025 (primary parent): *“you can wrap them in cotton wool too much. I think they need to be made aware….what’s bad and what’s not….”*

PA105 (secondary parent): *“….She’s got to have small risks...got to be able to cope with…risks…if something happens, know that she can deal with them ….”*

## Discussion

### Key findings

In-depth interviews with CYP and parents were undertaken to explore whether future well-becoming was considered important to CYP’s quality of life during childhood. Findings suggest that CYP aged 6–15 years and their parents are concerned with future well-becoming now, as factors such as future achievement, financial security, health, independence, identity, and relationships were identified as key to future quality of life. CYP discussed well-becoming mostly in terms of things that they wanted for their futures and indicated that they wanted to aspire towards particular outcomes. These were typically positive aspirations, although some CYP expressed concerns around future financial security. These aspirations and concerns were also mirrored by the parent informants, and many well-becoming themes raised by parents were expressed in the importance of CYP realising an aspect of wellbeing now to ensure a related well-becoming aspect in the future. This included engaging in healthy behaviours during childhood to encourage the continuation of these behaviours into adulthood and developing identity and independence, which parents suggested would facilitate future capabilities. Parents thought that establishing positive relationships in childhood would lead to positive future attachment and emotional security. The importance of achieving educationally now to facilitate access into selected career paths and future financial security was raised by CYP and parents alike.

A greater number of well-becoming themes were raised by parents and discussed by proportionally more of the parent sample; however, future identity, attachment, financial security, and achievement were also introduced by CYP. Factors related to future financial security, future attachment and identity were mentioned mostly or solely by secondary-aged CYP. Future financial security was also only discussed by secondary-aged parents. The focus on these well-becoming topics in the secondary parent and CYP groups may be due to milestones such as buying a house or having a family seeming more imminent than in the primary aged groups. Expressions of future identity from secondary CYP in discussing future career options may be due to emphasis from schools on decisions for subjects of study and future career choices. However, it seems likely that other personal characteristics factored into why particular informants consider certain well-becoming factors important. For example, the majority of CYP who were concerned by future financial security came from the most deprived socio-economic backgrounds, and this was true across both the primary and secondary CYP groups. Factors such as the CYP’s gender, ethnicity and family background did not appear to impact well-becoming factors raised.

### Strength and limitations

A strength of this study is that it provides an exploration of concepts important to CYP’s well-becoming and aspiration, and their links to CYP’s current wellbeing [21], from the perspectives of both CYP and parents. Further strengths were the large sample size, and the use of direct research with CYP, adopting a “rights-based approach” whereby the child’s voice has been prioritised and used [16, 32–34]. However, the study has limitations. The CYP sample could have benefited from greater representation, including from CYP living outside of England and from rural areas in both groups, and those from the most deprived backgrounds in the primary group. There was also less representation from parents of CYP from lower socio-economic and non-White British groups. Whilst the study inclusion and exclusion criteria aimed for a broad a sample of CYP and parents, we cannot rule out the possibility that in some families where both the adult and CYP were communication impaired, they may have wanted to take part in the study but were not able to. The study is focused on the views of school aged CYP aged 6–15 years, and thus, it would be important to investigate whether findings are also applicable to 0–5 years and 16–18 years age groups. Having said this, a maximum variation approach to sampling was taken to enhance representativeness as far as possible, and analysis picked up differences between different informants in responses, for example, that CYP from the most deprived backgrounds were most concerned about future security. The analysis also included the perspectives of parents, who typically advocate for their children.

The shift from in-person to online recruitment and data collection did not appear to impact the richness of the interview data and actually facilitated the recruitment of CYP and parents from further afield. However, conducting interviews online did make it more difficult to monitor parental involvement in CYP’s interviews, potentially impacting the topics that the CYP discussed.

### Comparison to existing literature

To the authors’ knowledge, our study is unique in empirically exploring the importance of well-becoming factors to CYP’s current quality of life, with a view to considering whether it could be important to incorporate future factors into outcome measures for CYP. However, the findings of our study align with Hart, who reported that CYP aged 15–19 years commonly mentioned five aspirations that were important to them, including making a difference, job satisfaction, status, personal happiness, and wealth [20, 35]. Although Hart’s (2012) research was in an older population than in this study, there appears to be overlap between the important items suggested by CYP in both studies, specifically those related to future security in terms of wealth and status (i.e. having a good job). Making a difference appeared related to CYP’s discussion of developing identity and future career choice. However, our study identifies well-becoming factors beyond those related to education and employment and highlights issues such as future relationships, health, and independence. A study by Allard and colleagues (2014) similarly found that independence and achieving future aspirations were important to CYP and parents of CYP with neurodisability, with the qualitative findings demonstrating that parents particularly valued interventions which supported CYP to achieve their potential [36]. Our findings add support to existing literature which emphasises the importance of investing in children’s futures to facilitate opportunities for future flourishing [14, 20, 37, 38], with the CYP and parents in our study suggesting that they found it both relevant and important to aspire for particular outcomes in the CYP’s futures and that they considered these outcomes to be important to future quality of life. Our study findings further fit with literature suggesting that investment in future well-becoming could be increasingly important for groups of CYP who may face inequality in terms of their future capabilities [38, 39], as parents and CYP from the most deprived socioeconomic backgrounds were most concerned about their future financial security.

### Implications for the development of child measures

Since undertaking the qualitative work looking at the importance of well-becoming for CYP, we have completed the development of two capability wellbeing outcome measures for CYP aged 6–15 years (split into two measures for primary school aged (6–10 years) and secondary school aged (11–15 years) CYP). To incorporate well-becoming into these measures, we decided to (1) ensure that factors important to well-becoming were represented in the wellbeing attributes (work to develop the wellbeing attributes will be published shortly), but also (2) to explicitly include a well-becoming attribute in each of the measures. The wellbeing attributes focus on the capabilities that CYP and parents identified as important to current wellbeing, but also incorporate factors which have the potential to impact future well-becoming, for example, through the inclusion of an “achievement attribute” which can capture the impact of interventions on CYP’s capability to achieve now, but also assume that improving this capability now could lead to continued capability to achieve on an ongoing basis. This is grounded in the notion from parent informants that realising an aspect of wellbeing now could lead to a related well-becoming aspect in the future.

**Fig. 1:**
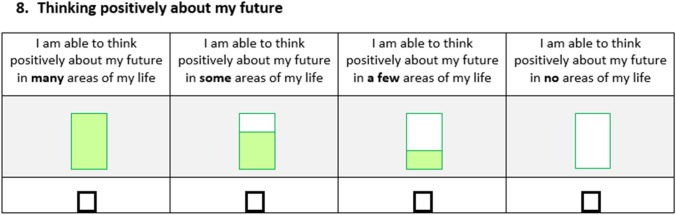
Well-becoming question from 11 to 15-year-old capability wellbeing measure

However, based on our research findings and discussions in the literature about the importance of CYP having positive aspirations for the future, we decided that it was also important to distinctly measure the impact of interventions on future wellbeing. CYP suggested during interviews that they valued thinking about their futures in a positive way and thus the well-becoming attribute in each of our measures focuses on CYP’s “capability to aspire” and asks about whether CYP are able to think positively about their futures across multiple areas of their lives. The question reads *“thinking positively about my future”* and gives the options “*I am able to think positively about my future in ****many**** areas of my life, “I am able to think positively about my future in ****some**** areas of my life, “I am able to think positively about my future in ****a few**** areas of my life* and *“I am able to think positively about my future in ****no**** areas of my life”* (see Fig. 1 for the question as it appears in the 11–15 years questionnaire). The benefit of having a separate well-becoming attribute is that we can distinctly capture the level of positivity that CYP are able to feel about their capability to meet their aspirations and achieve future outcomes (whatever those may be) and thus the impact of interventions to raise aspirations. In terms of child measurement, this allows us to specifically measure the outcomes of programs or policies designed to improve CYP’s motivations and ambitions for the future, which has been highlighted as crucial to encouraging CYP’s future wellbeing and to reducing inequalities [19, 20]. This is just one way of adding wording to reflect aspiration/well-becoming in relation to the development of a capability wellbeing measure. For researchers who are interested in capturing this aspect of children’s outcomes in other contexts, other types of wording might be more appropriate and could be explored.

In the context of economic evaluation, we can direct resources towards interventions which demonstrate such improvements, potentially providing a more cost-effective way of promoting wellbeing throughout the life cycle, as resources are directed to address inequalities and constraints on future ambitions early on, reducing the need for greater investment to reduce inequalities in adulthood [19, 35, 38]. An example might be investment in ‘social prescribing initiatives’, which aim to address the impact of the social determinants of health and improve health and wellbeing via community-based, non-medical interventions, such as arts and education schemes [40, 41]. There has been a recent focus on using social prescribing to tackle mental ill health in CYP [42] but community-based interventions could also have more wide-ranging impacts on CYP’s wellbeing [43] and potentially be used to develop and evaluate programs aimed at raising CYP’s future aspirations.

There are some potential limitations to the approach we have taken to include well-becoming in our measures, which may have broader implications for future measure development. In our measure for CYP aged 6–10 years, we decided to include the well-becoming question but to allow parents/guardians of CYP to complete it on their child’s behalf. This decision was based on feedback from parents in follow-up work which suggested that younger children might struggle to imagine their future capabilities in a meaningful way. We think that parents will be a good proxy for younger children in terms of interpreting and articulating CYP’s level of future aspiration; however, it may be considered a limitation that CYP are not completing this question directly. Including well-becoming attributes also has the potential for double counting, as there is potential for future wellbeing outcomes to be captured by modelling wellbeing over a CYP’s lifetime. Such issues might offer support to including well-becoming in CYP measures by developing attributes which mutually incorporate factors important to current wellbeing and future well-becoming, with the assumption that improvements in wellbeing in a particular area will have an ongoing impact on future well-becoming outcomes in related areas. However, it is only by including explicit indicators for well-becoming that we can be sure that we are capturing information on interventions which can more directly impact CYP’s future outcomes, such as through a focus on assessing whether interventions raise the future aspirations of CYP. This said, further research would be required to ascertain the degree of any overlap between what is being measured by wellbeing attributes in relation to an attribute covering well-becoming. This could be done through quantitative validity work [44] to explore the associations between CYP or parent responses to a well-becoming attribute in relation to others, and through qualitative ‘thinkaloud’ work, to understand what CYP or parents are thinking about when they complete each attribute within a measure.

## Conclusion

The findings of in-depth interviews with CYP and parents suggest that well-becoming factors—or being able to think positively about outcomes in CYP’s futures—are important to CYP now. Such evidence, taken alongside literature which promotes investment in CYP’s futures during childhood, suggests that it could be important to include well-becoming outcomes as part of health and wellbeing measures for CYP, with the aim of enhancing CYP’s future opportunities by investing directly in factors considered important to future wellbeing. In the context of our own work to develop capability CYP wellbeing measures, we have demonstrated the inclusion of a well-becoming attribute, specifically measuring CYP’s ‘capability to aspire’, with the hope that this indicator can facilitate investment in interventions which raise CYP’s future aspirations and thus can more directly improve their future outcomes. We hope that the evidence and ideas generated by our study findings and CYP measure development might be helpful to other researchers thinking about whether and how well-becoming indicators could be incorporated into their own measure development contexts.

### Supplementary Information

Below is the link to the electronic supplementary material.

Supplementary file1 (DOCX 1227 kb)

Supplementary file2 (DOCX 38 kb)

## Data Availability

We have included all empirical data relevant to this publication in the supplementary information provided.
