# Supplementary material for: Is well-becoming important for children and young people? Evidence from in-depth interviews with children and young people and their parents
Source: Qual Life Res. 2024 Jan 31;33(4):1051–61. doi: 10.1007/s11136-023-03585-w (PMC10973085; doi:10.1007/s11136-023-03585-w)
Supplement: Supplementary file 1 — Supplementary file1 (DOCX 1227 kb) [file 11136_2023_3585_MOESM1_ESM.docx]

**Appendix 1 – Topic guides for interviews (interview questions)**

1. **Questions from topic guide for CYP interviews**

Questions about the drawing task (attributes for measure):

- Can you tell me about what you have drawn/written (*go through the items on A3 paper one by one*)?

*Probe:*

- - Why is [item] important to you?
  - Why do you like it? Why does it make you happy? What is it about it that is important to you?
  - You have put [item A] as more important to you than [item B], why is that (continue for all items)?

Questions about the future:

- What would you like to do in the future?
- Where do you see yourself in the future? What will you be doing? What will you have?
- Are there any other things that you would like to do/achieve?

1. **Questions from topic guide for parent interviews**

- What things do you consider to be important to your child’s quality of life?

*Probe:*

- What is it about these things that makes them important?
- Which of these things do you think is most important? How do they compare?
- Is there anything that you think could improve your child’s quality of life?
- Anything that you are not so happy with?
- Things that you would like more/less of in your child’s life?

Questions about child/young person’s future:

- What would you like for your child in the future?
- What things do you consider important to your child’s future happiness?

**Appendix 2 – Example of completed hierarchical mapping activity**

**
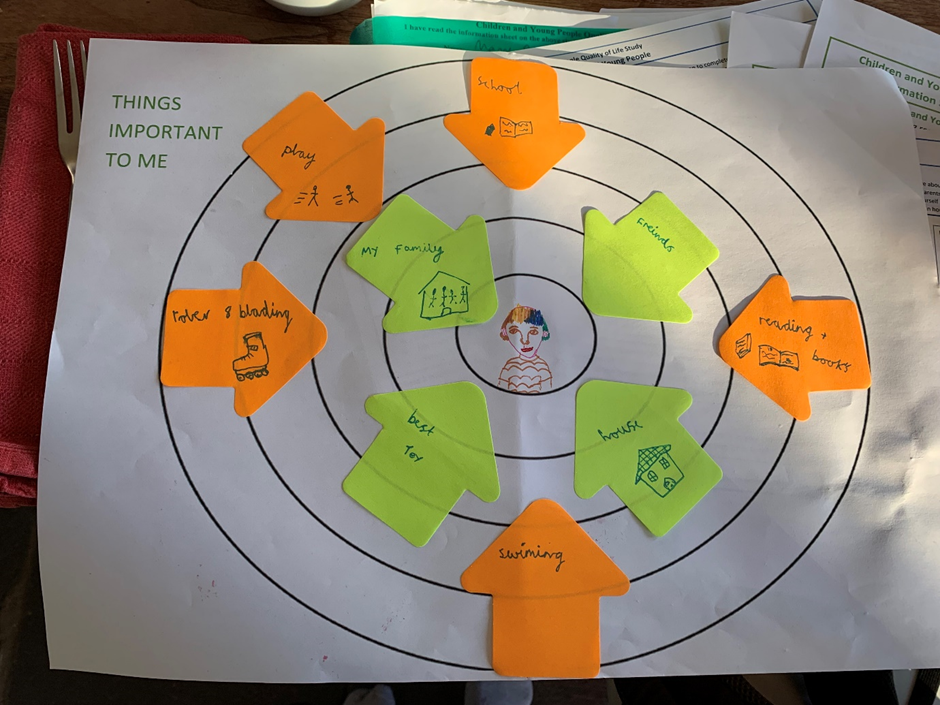
**
