# Supplementary material for: Is well-becoming important for children and young people? Evidence from in-depth interviews with children and young people and their parents
Source: Qual Life Res. 2024 Jan 31;33(4):1051–61. doi: 10.1007/s11136-023-03585-w (PMC10973085; doi:10.1007/s11136-023-03585-w)
Supplement: Supplementary file 2 — Supplementary file2 (DOCX 38 kb) [file 11136_2023_3585_MOESM2_ESM.docx]

**Supplementary Information**

**Article title:** Should health and wellbeing measures include well-becoming elements for children and young people? Evidence from in-depth interviews with children and young people, and their parents.

**Journal name:** Quality of Life Research

**Author names:** Samantha Husbands, Paul Mark Mitchell, Philip Kinghorn, Sarah Byford, Cara Bailey, Paul Anand, Tim J. Peters, Isabella Floredin, Joanna Coast

**Corresponding author:** Dr Samantha Husbands, Health Economics Bristol, Population Health Sciences, Bristol Medical School, University of Bristol, BS8 1NU. Email: [Samantha.husbands@bristol.ac.uk](mailto:Samantha.husbands@bristol.ac.uk)

**Description of supplementary information:** Qualitative data to support the findings of the research contained within this article (all quotations from informants which are related to the importance of well-becoming to children and young people’s wellbeing)

**Primary aged children and young people**

**Achievement now for the future:**

PC004 (primary CYP): “*To get an education’, because everyone, like, because if you don’t get an education, you’re more likely to be like, and you won’t be able to, like you need an education to be successful in life.”*

PC004 (primary CYP): “*it’s just what we need to get, so- so you can get- if you don’t get an education, you’re more likely not to get like money and you won’t be able to pay something in the future, yes, and stuff like that.”*

PC005 (primary CYP): *“…academically my education is really important because it depends what job you’re wanting, what work you want to do…”*

PC005 (primary CYP): *“Because erm the school that I am going to, they are going to look at them, so they are going to look at our SATs results and they are going to divide us into like groups like of people that go like really high, people that go for than the average, the average, below average and the really low. Yeah and that’s something that would stay with you for the rest of my life and yeah.”*

PC005 (primary CYP): *“If you go to like a not as good, an unrecognised university, the education isn’t as good as others and it will. If you go to the best university as possible that will get you a better job.”*

PC005 (primary CYP): *“Because erm like education it affects everything you do like erm if you want to become something when you’re older it’s education that will be supportive, you can’t be like “I’m going to be this” and not have any education at all. So what I say is like erm education leads you to a good school, a good school leads you to a good secondary school, a good secondary school will lead you to a good sixth form or college, and that will lead you to a good university and that will lead you to a good job. So will help you on in life.”*

PC005 (primary CYP): *“Like a good job. A good placing job. Yeah so most people who did not get a good education, they end up doing really, I’m not going to say bad jobs, but poor jobs.”*

PC005 (primary CYP): *“just to be successful….Get a good job. Get a, er go to a good university and er get a job that is suitable for me”*

PC021 (primary CYP): “*We’ve got to learn, otherwise… Like reading, you’ve got to be able to read when you’re older otherwise yes… You’re reading all the time. You need to learn maths because, if you’re a shopkeeper, you’ve got to do quite a lot of maths.”*

PC023 (primary CYP): *“I’m going to pass university and everything else…And have a good job, yes…Like, I don't want to mess about and be poor. I just want to have a good job and feel happy.*

PC027 (primary CYP): *“because if you don't do school work, well first of all you'll get told off, second of all you need an education. If you don't do all this, you won't get an education, job or anything like that. That's important about life. If you don't get any of that, it's mostly your fault because you didn't do any of your work in school. School is the key to life….I've always wanted to be an author.”*

PC039 (primary CYP): *“Secondary school. You need to learn, so in secondary school, Year 9, Year 10, GCSEs and exams and that stuff, you need to know it…You need to keep it all in your brain so when it comes to all that it's in there already and you can just let it all out for the tests…. [it impacts] Where you're going to work. For example, if you work in a bank, then you need to know maths for money, mental strategies, and college. Yes, and jobs.”*

PC092 (primary CYP): *“...if you didn’t know anything then you wouldn’t be able to get a job and earn money and then you wouldn’t have a house.”*

**Future physical and emotional security:**

PC013 (primary CYP): *“Probably my mum and dad getting a job, it would make it much easier, because we don’t have much money….When they get a job, it would make it easier because we’ll start getting money then, and then we can start to pay for stuff fun, the fun stuff, and toys and that. Now, we can’t, because we don’t have much money. I’m really sad about that. One day, we’re just going to run out, so we can’t pay our bills and stuff.”*

PC098 (primary CYP)*: “Then my last one is getting a good job when I’m older….Like getting a job that I enjoy, while gets me quite a lot of money….So, you can get a nice house…..So, you have somewhere to live.”*

PC092 (primary CYP): *“...if you didn’t know anything then you wouldn’t be able to get a job and earn money and then you wouldn’t have a house.”*

PC023 (primary CYP): *“I don’t want to be poor, then I’d live in the streets. I don’t want to.”*

**Future attachment:**

Interviewer: “*Okay. And what do you think will make you happy [in a future job]?”*

PC023 (primary CYP): **“***Having friends in the job.”*

**Future identity:**

PC039 (primary CYP): “*I really want to be a nurse…It's just interesting learning to be a nurse, training how to be a nurse. A nurse or a doctor, learning about the body…. The right type of medicines to give to people, what's inside the medicine. Also, talking to people about if they have a problem and just helping them. Yes.”*

**Secondary aged children and young people**

**Achievement now for the future:**

PC024 (secondary aged CYP): “*School is important to me because if I go to school, in later years, I can have a really good education and that thing, yes.”*

PC024 (secondary aged CYP): “*For example, my future, it could really help me and, yes…It could help me, for example, like I can get a good job. I can do so many things, yes, which will help me with my future, and I could go to university and everything, yes, yes…. It’s because it can help me get jobs and anything really easy, yes.”*

PC034 (secondary aged CYP): *“Well, if you go to school, you can have a good education and with a good education you can use that in the world that gives you more choices when you’re older. If you don’t go to school, you won't get what you wanted, or some things that you wanted.”*

PC042 (secondary CYP)*: “Because then if you do the subjects that you like and the subjects that you might want to study in university, you’ll get a better grade in university and then you’ll have a chance of getting the job that you want….then I can do what I like”*

PC042 (secondary aged CYP): *“Because if you don’t have a good education, you won’t get the job that you wanted. And if you don’t have the job you want you won’t succeed in life.”*

PC043 (secondary aged CYP): *“Say you want to get a job, you have to have certain degrees and stuff so going to university would help you do that.”*

PC043 (secondary aged CYP): “*Because if you want the job you want to get, say you wanted to be a professor, you’d have to have degrees and stuff. Going to university will help you get it otherwise you can’t get a degree.”*

PC106 (secondary aged CYP): *“The field of work I want to go into, you can’t get into it without school.”*

**Future physical and emotional security**

PC034 (secondary aged CYP): “*I’ve heard so many stories of like people not being able to afford houses right now because of inflation and stuff, but yes, I just hope that I’ll be able to afford a house.”*

Interviewer: “*Why do you think it’s important to have a good job”*

PC043 (secondary aged CYP): “*Because then you get money and you can feed yourself and look after yourself….As long as you have food and a home, that’s really all you need.”*

PC046 (secondary aged CYP): *“I want to go to a good sixth form and university….Because I would like to get a stable job. Yes….I want to be, I suppose, a professor or a doctor or something.”*

**Future attachment**

PC043 (secondary aged CYP): *“When I’m older I’m probably going to adopt some kids because I think if you adopt kids, you’re giving them a home rather than leaving them there…”*

PC043 (secondary aged CYP): *“But I think I’d want to stay close to home…. Because then if I ever needed anything I could easily get it, whereas if I was in America or something, I couldn’t just be like, “Mum, can you bring me this?”…”*

PC109 (secondary aged CYP): *“Because as well as getting your education and giving yourself opportunities in the future, it [school] gives you social skills. If there are 80 people in your year, then you are going to make loads of friends and then hopefully you will know them for the rest of your life.”*

PC113 (secondary aged CYP): *“[in the future] I always wanted a big house….but to myself or with my partner or something.”*

PC109 (secondary aged CYP): *“Getting married, having children…I want that…”*

PC111 (secondary aged CYP): “*...to have my own children. I’d like to settle down and have a family of my own….”*

**Future identity**

PC032 (secondary aged CYP): *“I want to be a doctor….Because it’s just about helping people and being a part of something….Like a community kind of thing.”*

PC106 (secondary aged CYP): *“I’d like to have furthered the cause of humanity in some way, be that building energy efficient cars with eco-friendly sustainable fuel cells or just helping a couple of people stay happy. I’d just like to be able to say that I have helped.”*

PC038 (secondary aged CYP): *“I really want to be a nurse…talking to people about if they have a problem and just helping them.”*

**Primary parents**

**Achievement now for the future**

PA008 (primary parent): *"I guess I am kind of aspirational for the children, yes….so [Husband] and I are quite clever, kids are quite clever, [CYP] especially is really bright for his age… I worry about him not having enough challenge at school, because he’s one of the eldest and he’s one of the brightest….”*

PA091 (primary parent): *“I think it’s important to teach children to try your best a lot of the time because I would want them to have a job- I wouldn’t want them to look back and think, “I didn’t try hard and I really wish I could have done this job but I can’t….””*

PA019 (primary parent): “*the educational attainment, it’s going to facilitate things for them that otherwise they won’t be able to access if they don’t know”*

PA025 (primary parent): *“It expands their minds and their brains, they learn stuff. Obviously, there’s no point, they have to do exams. It would be nice if they could get, then, a good job. That’s the way they need to…”*

PA089 (primary parent): “*Education. I do think education is important, personally. But I would never force her into that. Like I said before, I think it is important, education, I think it is important that she learns, that she goes- I personally would like her to go to university. She says she wants to be a vet, and I would love nothing more than for her to achieve that as a parent.”*

**Physical security**

PA025 (primary parent): *“I wouldn’t want them to be obese and cause health problems. I would like them to have a long life….”*

PA091 (primary parent): *“I suppose part of it is just learning self-discipline, isn’t it? It’s rules for as you get older I think, like go out, have fun, overeat a bit, drink some wine with your friends, that’s all okay. Don’t do that every night. Don’t do that all the time. Make sure that you’re just taking care of yourself as well because I think if you’re not healthy and you’re not fit, you’re not going to be as happy as you can be. I’m just helping them grow up to maybe learn how to have that balance to try and keep them healthy and happy.”*

PA011 (primary parent): *“So, we talked about trying to reduce sugar intake… laying down good habits early in life, both in terms of health and weight…ways of eating and cooking”*

PA014 (primary parent): “*I was brought up like that. My mum was really good at home cooking and stuff, she drummed it into me healthy eating and everything. I suppose I do feel like that’s important. The whole five a day. I feel like setting patterns early on works really well.”*

PA017 (primary parent): *“I read somewhere that if they do gymnastics when they’re little, even if they don’t continue to do it throughout their lives, it just makes their joints more open and more able to do things as they get older….Like as their bodies are growing and developing. So I think just for future health in their body…If they’ve had something they’ve loved doing when they were wee, then maybe that will be something they love when they’re older, or they’ll just be conscious that they like to move their body.”*

**Future attachment**

PA014 (primary parent): *“…To be happy…. Relationships that are positive and fulfilling, whatever relationships they choose”*

PA011 (primary parent): *“[friendship] gives him confidence about himself and it gives him social skills. And practising social skills, really, with the safe people that are in his life….”*

PA017 (primary parent): *“[friendship] is important to them growing up so they can have different friendships and understand the different needs of different people. Have empathy with other’s needs and things, yes.”*

PA089 (primary parent): *“Because I think it’s right for her, as well, to know what positive relationships are….I want her to know that cuddling and being warm and affectionate to somebody, or someone doing that to you, is important. Because it makes everyone feel nice, when you have a nice cuddle from someone that actually means it. But I don’t want her to think that, if she gets into an abusive relationship, that that’s normal and that’s okay, and that’s what love is.”*

**Future identity**

PA019 (primary parent): *“I would really like them to be confident women who have a clear idea about what they want to do and are, in a way, able to aim for that…..”*

PA011 (primary parent): *“….just all those little things add to that sense of purpose in life, that you feel passionate about something, you really enjoy something, and that’s what makes you tick.”*

PA014 (primary parent): *“Just them thriving within what they want to do and their areas of interest and stuff. They hopefully end up with a job that they’re happy with and they feel fulfilled in”*

PA008 (primary parent): *“I’m really happy if they want to go off and learn new things and get new experiences, I suppose….I think it’s just helping them to know where their interests lie and what they like, so [child] has done [instrument 1], [instrument 2] at school, so she did that and then she had some ukulele lessons at home and then she moved up because it got too easy so she did [instrument 1], and then she’s moved to [instrument 3], now she wants to do singing lessons when she’s at school. And I wasn’t sure about the singing lessons, I would rather that she learned a different instrument, but she really wants to do it, and I think it’s nice to have that opportunity to try those different things and really kind of find her identity, I suppose.”*

**Future independence**

PA025 (primary parent): *“you can wrap them in cotton wool too much. I think they need to be made aware. It depends on the age, obviously, but they’re going to… Friends are going to talk about things, about stuff, they’re going to see things on telly, it’s just… As long as they are aware what’s bad and what’s not, and it’s not too bad. They’ll have a better understanding…”*

PA089 (primary parent): *“Because I think, scary as it is, she’s 10, she’s 9, coming up 10….And I think, growing up, she needs to have that responsibility. Because it’s not long before we’re going into the big bad world and then she’s got to manage her life and be responsible.….But I think it’s important, while I’m here, to support her with that and to guide her, that she gets that bit of freedom, so that she can- Maybe, hopefully, it will help her later on in life with her management of her life.”*

PA019 (primary parent): *“I don’t closet them too much. They’re all quite physical, like climbing and stuff like that. Obviously, try and prevent anything serious, but I won’t be like, “Don’t go on that.” I want them to experience stuff and if they slip and hurt themselves it’s like, “That’s really hard, but now you know not to climb on that next time.””*

PA091 (primary parent): *“I don’t want them to just think, “Well what mum and dad want isn’t important because they had us and we’re the most important.” I don’t want them to think that the world revolves around them. I don’t want them to think, “Well that’s okay because mum and dad will just sort it out because they’re always free.”*

**Secondary parents**

**Achievement now for the future**

PA033 (secondary parent): *“education is really important to me, and yes, I wouldn’t have chosen a school where I didn’t think she was going to get a good education…So, to get GCSEs and A levels and have the options for the future….you have more doors open to you if you have a degree…I’ll definitely encourage her to study, up to that level….”*

PA037 (secondary parent): *“…and we do reinforce that “If you go to school and you do well, there would be a good job after it. So, there is a lot of importance placed on school and education.”*

PA097 (secondary parent): *“Ultimately, that is an expectation in our family, that they will work hard, they will go to university…. But just to know that the intention is that they will work hard, and they will get a good job hopefully…”*

PA102 (secondary parent): ***“****Because it gives them the goal they want to achieve; it gives them that. Say, for example, they want to go into… My daughter wants to do a dessert shop, she wants to be able to sell cakes and sell milkshakes. If you have a barrier there then they lose opportunities to get that job that they want….it’s difficult for them to go ahead and do it I think.”*

PA100 (secondary parent): *“I think school is about opportunity…. School gives you opportunities to do things…. [CYP] is talking about being an archaeologist, so she’s chosen GCSEs that will enable her to do that….”*

PA031 (secondary parent): “*Obviously, education. I’ve made that clear, how important that is. I’d go to any lengths to make sure they have a good education. They both want to be doctors, but in different fields, the two that are close in age. I’d like to see that happen. I’m a great believer that you don’t have to be a stereotype of where you’ve come from and where you live. You can achieve anything if you want it.”*

PA112 (secondary parent): *“….academic success is prized in our household, you know? We're super proud of their achievements and of what they do and they've all grown up believing that that's what's expected, that's an academic pathway…”*

PA031 (secondary parent): “*It’s just your stepping stone into life, isn't it? I think it’s so much harder, now, to get a job. There were days you could walk out of one job and into another. Now, there are so many people going for the same job. They might have something that you haven’t. I just really hope they do well.”*

**Physical and emotional security**

**Physical security (financial and health)**

PA097 (secondary parent): *“…. It’s just being able to have the money to be able to live comfortably, isn’t it? To have a roof over your head. To be comfortable….”*

PA107 (secondary parent): *“Because they do swimming, and it is obviously such a good sport for them, and you can see in front of their eyes that they're growing nicely. We do our best with food, but we are a working family, so there are days when it’s oven chips and things like that. But there is always fruit around….they understand balanced plates, calorie control and that sort of thing. But for us, it’s just that they are growing to be strong, healthy girls.”*

PA031 (secondary parent): “*just living in [location], the house prices are ridiculous. To even get on the housing ladder, you’ve got to be a pretty good earner to get a decent mortgage….”*

PA031 (secondary parent): “*I think outside factors do matter, where he ends up and financial burdens and things like that. I’m hoping that I can help, as much as possible, to ease any of those factors. It’s so expensive, I don’t know what’s going to happen down the road. I do save for them every week, into their savings accounts, to give them a little nest egg to start them off. Hopefully… I doubt it will be enough for a deposit on a house. Hopefully it will be driving lessons or help towards university costs or…”*

PA033 (secondary parent): *“We’re really lucky because my mum came into lots of money when my gran died. So, we’re never going to be destitute, you know? We’re financially safe, and we’ve been saving towards her college fund, should she need to go to college, and all that kind of thing. So, we’re actually in a really good position. We’ve got a house that we rent out as well. So, she is… I’m not going to say to her, “You’ll be fine.” Because it’s probably quite good to be thinking you’ve got to support yourself, but actually, she’s never going to be in a position where she is going to be on the streets, do you know what I mean?”*

PA036 (secondary parent): “*I don’t know, it’s hard really. We seem to be in such a period of change at the moment. I think financial, and job, security are going to be important to him, particularly in the long term. There are so many jobs, now, that are zero hours contracts and all that kind of thing. I’m really worried for young people, how they’re going to have full careers and things like that.”*

**Emotional security**

PA112 (secondary parent*)****:*** *“I think if you've tested out and you know what strong bonds are and strong links are, then that gives you the confidence in any new relationship - be it a romantic relationship or just a professional relationship - to show all the things that are important in good relationships, flexibility and compromise and things like that. You know, being in a big family and being used to being in this secure family really gives you that grounding*.*”*

PA037 (secondary parent): *“Social relationships are important, you know, to have that confidence to be able to go out and speak to people. We’re lucky enough to have really good neighbours….So, we go out and we get to meet all the neighbours and things like that. It’s important for them to feel a part of society, and to feel that they fit in somewhere. Otherwise, that could lead to them being a bit reclusive and not mixing, and leading a bit of a sheltered life.”*

**Future attachment**

PA110 (secondary parent): *“So she has peers, people her own age that she can relate to and that they can do things together and hopefully those friendships will last well into her adulthood. They'll have memories together. They'll be able to think about and talk about things, have reunions and things. It's really lovely to have that.”*

PA110 (secondary parent): *“have good relationships, have a lot of people around her, have a good support network. Yes.”*

PA100 (secondary parent): *“to have enough love and enough friendship in your life to enjoy what’s going on and to have enough people to support you, I think that’s really important.”*

PA107 (secondary parent): *“they're good friends. I don’t expect them to live in each other’s pockets at all but… you just know that you’ve always got your sibling there for you. Because obviously, at some point, we won’t be here for them.”*

PA112 (secondary parent): *“…Seeing how he is with his family, I think it'll be important to him to have a good circle of friends and still close ties with his family and, obviously, his own family in time.”*

PA110 (secondary parent): *“When they become adults they're going to be going and working in different places with all different sorts of people. I think it's really good to already be part of a diverse group to respect different backgrounds, different people, and have tolerance I suppose as well...”*

PA107 (secondary parent): *“…obviously she is now getting a group of friends obviously at secondary school, she has got some friends left from primary school. So, she is having to learn how hard it is to work to keep friendships going when you're not together every day.”*

PA105 (secondary parent): *“She’s made some really good friendships, lovely people that she’s met. She’s had challenges with a couple of friends in the past but that all helps build, doesn’t it...Yes, I definitely think a network of friends gives you a very different type of support. Some break down, some don’t work, but it’s a learning curve and it’s all an experience.”*

PA037 (secondary parent): “*So, getting married and settling down, having a family themselves, that just seems like something should occur naturally. So, I hope that they do achieve that, as well.”*

**Future identity**

PA100 (secondary parent): *“As a child, that’s really important. You should have a go at lots and lots of different things and then discover what you actually like. If you don’t have a go at stuff you’ll never know whether you like it or not. He [CYP] might came back to it in 30 years and be an old man in his shed with his woodworking tools and talk about when he was 11 he was bought a plane….”*

PA110 (secondary parent): “*Just so they can have the opportunity to have experience of lots of different subjects, lots of different activities, sport…and really find out what they are good at so they can make better decisions for their future when they become adults. Find out what they really enjoyed, what they would like to enjoy and learn more of.”*

PA112 (secondary parent): *“….I think it's just part of being your own person and having your own strong identity that you carry through life. You should be proud of where you come from, you shouldn't be embarrassed or ashamed of it…..I think it's really important that you are proud of your roots and your heritage and the links that go back””*

PA036: *“…He’s got to decide what his sexuality is or whether he gets married, all those kinds of things. I don’t have a vision of what we’re trying to turn him into beyond somebody who can make his own decisions and feel comfortable with who he is, that’s where I come at I think.”*

**Independence for the future**

PA036 (secondary parent): [*discussing her son going out and meeting friends independently] “…. Part of it is a bit of responsibility, just being out there by yourself and having to take the consequences of how you act in public and around other people…. There is a bit about taking some independent decisions, I think, over what they do.”*

PA097 (secondary parent): *“…. I just think in any walk of life, they’ve got to be able to be confident. They’re not always going to have someone there to be able to do those things for them.…”*

PA105 (secondary parent): *“….She’s got to have small risks...got to be able to cope with…risks…if something happens, know that she can deal with them ….”*

PA100 (secondary parent): “*Things happen, whether you miss the bus or you get shouted at by an adult or you’re late home or something happens, and you know that you have the capacity in yourself to deal with that. That’s really important for me, that they learn that skill. It’s about putting him in situations where he feels slightly uncomfortable but he comes away thinking, “You know what, I dealt with that.” I think that will see them through to adulthood in a really great way.”*
